# Structure–property relationship of a complex photoluminescent arylacetylide-gold(I) compound. I: a pressure-induced phase transformation caught in the act

**DOI:** 10.1107/S2052252524007681

**Published:** 2024-08-23

**Authors:** Róża Jastrzębska, Tomasz Poręba, Federico Cova, Daniel M. Tchoń, Anna Makal

**Affiliations:** a Biological and Chemical Research Centre, Faculty of Chemistry University of Warsaw Żwirki i Wigury 101 02-089Warszawa Poland; b Laboratory for Quantum Magnetism, École Polytechnique Fédérale de Lausanne, CH-1015Lausanne, Switzerland; c ALBA Synchrotron BL31-FaXToR, Cerdanyola del Vallés, Cataluña, Spain; d Molecular Biophysics and Integrated Bioimaging Division Lawrence Berkeley National Laboratory,1 Cyclotron Road Berkeley CA 94720 USA; Formby, Liverpool, United Kingdom

**Keywords:** crystal engineering, phase transitions, polymorphism, properties of solids, materials science, high-pressure diffraction

## Abstract

A pressure-induced triclinic-to-monoclinic phase transition has been caught ‘in the act’ over a wider series of high-pressure synchrotron diffraction experiments conducted on a large, photoluminescent organo-gold(I) compound. Here, we describe the mechanism of this single-crystal-to-single-crystal phase transition, the onset of which occurs at ∼0.6 GPa, and we report a high-quality structure of the new monoclinic phase, refined using aspherical atomic scattering factors. Our case illustrates how conducting a fast series of diffraction experiments, enabled by modern equipment at synchrotron facilities, can lead to overestimation of the actual pressure of a phase transition due to slow transformation kinetics.

## Abstract 2

A pressure-induced triclinic-to-monoclinic phase transition has been caught ‘in the act’ in the course of a wider series of high-pressure synchrotron diffraction experiments conducted on a moderately photoluminescent gold(I) compound. Our experiments illustrate how conducting a fast series of experiments, enabled by modern equipment at synchrotrons, can lead to an inaccurate estimation of the actual pressure of a phase transformation.

## 1. Introduction

Application of increased pressure provides a unique means of control over the intermolecular interactions in crystals without changing the chemical composition (Valiente *et al.*, 2016 ▸). This in turn opens possibilities to discover new solid phases and construct phase diagrams of various substances (Ravindran *et al.*, 2019 ▸; Tse, 2020 ▸; Vervoorts *et al.*, 2021 ▸) but also to systematically study properties of materials such as photoluminescence (Li *et al.*, 2022 ▸; Tong *et al.*, 2022 ▸; Zou *et al.*, 2024 ▸).

With the recent advent of fourth-generation synchrotron sources, pixel-counting detectors, membrane diamond anvil cells (DACs) and dynamic compression systems, high-pressure research at large facilities has become ever faster, more comprehensive and more efficient (Donath *et al.*, 2023 ▸; Poręba *et al.*, 2022 ▸; Pascarelli *et al.*, 2023 ▸). Strong incident beams coupled with automated pressure control offer full single-crystal X-ray diffraction datasets in a couple of minutes (Guńka *et al.*, 2021 ▸; Ambach *et al.*, 2024 ▸), opening the possibility of tracking time-resolved processes (phase transitions or solid state reactions) hitherto only available for powder diffraction studies on simple, highly symmetric inorganic compounds (Husband *et al.*, 2021 ▸; Ricks *et al.*, 2024 ▸). Though such rapid nature of data collection is certainly welcome, it nevertheless demands heightened awareness about the kinetics of investigated materials.

A thorough review by Chandra Shekar & Rajan (2001 ▸) highlighted the impact of the pressurization rate on the actual path of oncoming phase transitions. Recent specific cases of inorganic materials (Husband *et al.*, 2021 ▸; Ricks *et al.*, 2024 ▸) as well as, on a different time scale, certain molecular crystals (Fisch *et al.*, 2015 ▸; Zhang *et al.*, 2021 ▸; Tchoń *et al.*, 2021 ▸; Yuan *et al.*, 2022 ▸) confirm that slow pressurization can result in very different transformation paths and observed phases. Most importantly, certain pressure-induced phase transitions characterized by slow kinetics may require hours, days or weeks to take place, and thus remain unobserved over the course of a rapid series of diffraction experiments (Szafrański & Katrusiak, 2017 ▸; Szafrański *et al.*, 2021 ▸).

As a part of our ongoing project aimed at relating crystal structures to physical properties in arylacetylide-gold(I) compounds, with a particular goal of elucidating the extent to which the crystal environment can affect their photoluminescence, we turned our attention to (pyren-1-oylacetylide)(triethylphosphine)gold(I). The compound is already known to produce at least two distinct crystalline materials, which differ in ligand arrangement, the presence/absence of the aurophilic interactions and photoemission properties (Głodek *et al.*, 2018 ▸). Its crystal form, originally named 1a′, here denoted pyrEt-α, which could be represented as (pyren-1-oylacetylide)_2_Au⋯Au(triethylphosphine)_2_, is the focus of the current study due to the presence of aurophilic interactions. We conducted a few series of X-ray diffraction experiments with the aim of structure determination of this moderately luminescent material over a pressure range from 1 atm to 9.00 GPa and independently at temperatures from 90 to 300 K. These structural studies will be published separately together with the variable-pressure and variable-temperature analysis of the material’s photoemission, putting the latter in a wider perspective. Here we focus exclusively on the occurrence and the mechanism of a particular single-crystal-to-single-crystal (SCSC) pressure-induced phase transition occurring at a relatively low pressure of approximately 0.6 GPa. Our goal was to highlight how such transition can be tracked by means of single-crystal X-ray diffraction (SCXRD) even for a relatively large metal–organic system.

## 2. Experimental

### 2.1. Single-crystal X-ray diffraction experiments

The crystals of pyrEt-α were obtained according to the formerly published protocol (Głodek *et al.*, 2018 ▸). The structure of pyrEt-α has been redetermined under standard atmospheric conditions to serve as a reference for high-pressure studies. SCXRD data were collected using an in-house Rigaku Oxford Diffraction SuperNova four-circle diffractometer equipped with an Eos CCD detector and a molybdenum microsource (Mo *K*α, λ = 0.71 Å). The single-crystalline block was mounted on a Mitegen loop with a small amount of Paratone-N oil. Data collection and reduction were performed in *CrysAlisPro* (Rigaku Oxford Diffraction, 2019 ▸). Shape based absorption corrections were applied using the same software.

#### 2.1.1. Variable-pressure experiments

High-pressure SCXRD experiments were conducted at the ESRF ID15B synchrotron beamline (λ = 0.41 Å), beam size 4 × 4 µm, equipped with either Mar555 flat-panel amorphous Se detector (series I and multi-crystal approach) or EIGER2 X CdTe 9M detector (series II). The instrument model was calibrated with an Si NIST640b calibrant and a single crystal of vanadinite with known lattice constants.

The goal of the ‘serial experiments’ was to observe the diffraction pattern in a wide pressure range. Thin plate-like single-crystal specimens were placed in a symmetric membrane DAC of BX90-type using helium gas as a pressure-transmitting medium (PTM) (further details provided in Section S1 of the supporting information). The pressure was increased using a PACE6000 controller and verified *in situ* with reference ruby spectra (nominal estimated standard deviation on pressure determination 0.03 GPa). In the first series (I), four crystal specimens were placed in the DAC to gain better data coverage and compensate for radiation damage. These yielded a total of 29 useful datasets in the pressure range from 0.25 (3) to 9.03 (3) GPa, two of which, at a pressure of 0.60 (3) (crystal 2) and 0.65 (3) GPa (crystal 3) accordingly, will be commented on in more detail here. In the second series (II), a single-crystal specimen (crystal 4) yielded a total of 16 useful datasets in the pressure range from 0.25 (3) to 5.98 (3) GPa, two of which were collected 20 min apart at the same pressure of 0.82 (3) GPa.

#### 2.1.2. Multicrystal approach

In order to increase the reciprocal space coverage for the new phase being investigated and facilitate unrestrained refinement of its crystal structure, a total of five larger pieces cut out of a single crystal (crystal 5) of pyrEt were placed in a single DAC of a Merrill–Basset (MB) design in distinct orientations. The latter type of DAC provided a larger opening angle and easier loading, but as a trade-off it did not allow for gaseous PTM use. Paratone-N oil was used as a PTM, being the only liquid PTM on site that would not dissolve the compound under investigation. Initial rapid pressurization yielded 1.10 (3) GPa within the quasi-hydrostatic limit of the applied PTM (Klotz *et al.*, 2009 ▸; Tateiwa & Haga, 2009 ▸). Diffraction data were collected within 2 h of reaching this pressure. Data collection for each crystal specimen in each experimental series consisted of a single ϕ-scan in ± opening angle range (32 or 39° for membrane DAC and MB DAC, accordingly) with an angular step Δω of 0.5° and an exposure time of 1 s per frame. Each data collection took less than 5 min.

Data reduction was performed in *CrysAlisPro* (Rigaku Oxford Diffraction, 2019 ▸). Final datasets from the three largest pieces in the multi-crystal approach were merged using *SORTAV*, yielding the total data completeness of 71% up to a 0.65 Å resolution.

#### 2.1.3. Structure solution and refinement

Structures were solved using *SXELXS*/*SHELXT* and refined in *SHELXL* (Sheldrick, 2015 ▸) within the *Olex2* graphical environment (Dolomanov *et al.*, 2009 ▸; Bourhis *et al.*, 2015 ▸). The hydrogen atom positions were constrained to their closest carbons and had their atomic displacement parameters defined using the riding approximation. In the case of the multi-crystal approach, no further restraints or constraints were necessary. In the cases of limited data coverage (*i.e.* datasets collected with a membrane DAC), a number of chemically viable geometrical similarity restraints on C—C distances and atomic displacement parameters were applied during structure refinement.

The structure of the new monoclinic pyrEt-α phase at 1.10 (3) GPa was finally subjected to the Hirshfeld atom refinement (HAR) using *NoSpherA2*, an implementation of non-spherical atomic form factors in *Olex2* (Kleemiss *et al.*, 2021 ▸). The electron density was calculated from a Gaussian basis set single-determinant SCF wavefunction for a fragment covering twice the monoclinic asymmetric unit in the exact experimental geometry. Calculations were performed with *ORCA* (version 5.0; Neese *et al.*, 2020 ▸) using the DFT approach with *R2SCAN* functional, *x2c-TZVP* basis set and *DKH2* relativistic correction. The hydrogen atom positions remained constrained using the riding approximation. Molecular graphics were prepared using either *Olex2* or *Mercury* software (version 4.3; Macrae *et al.*, 2008 ▸). The data were deposited with the CCDC and assigned the deposition numbers 2360104, 2360105, 2360125 and 2360126.

## 3. Results and discussion

### 3.1. Structure of pyrEt-α at room temperature

**Figure 1:**
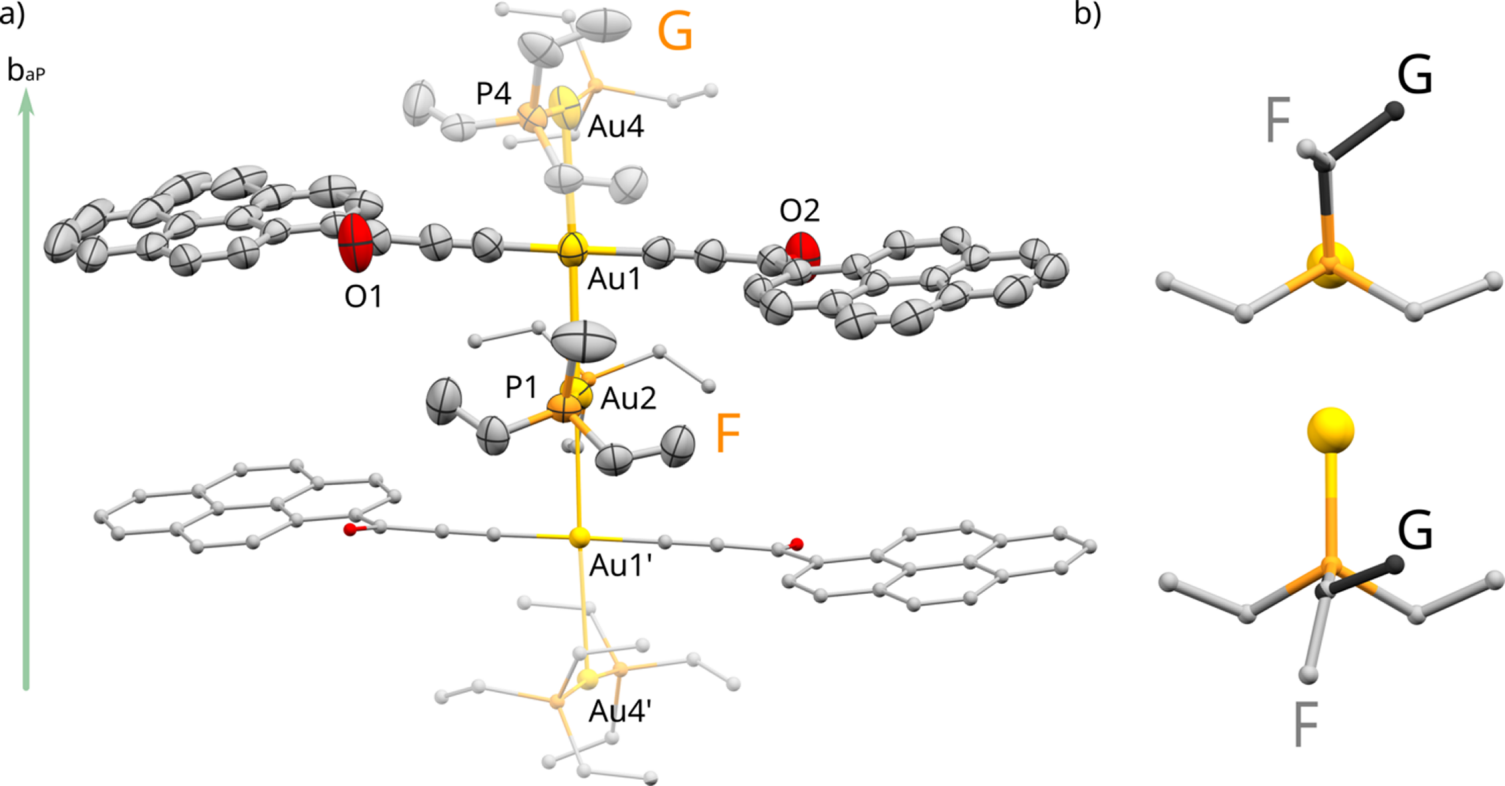
(*a*) Main building block of the crystal structure of pyrEt at 300 K: a rod of four Au atoms, disordered over two positions along its long axis. Atoms Au4 and Au4′ with their phosphine ligands represent the two alternative disorder variants, as the rod can begin at either ‘side’ along the [010] crystallographic direction. The parts with 50% occupancy are represented as semi-transparent. The atomic displacement parameters are presented exclusively for atoms from the asymmetric unit at 50% probability. Hydrogen atoms have been omitted for clarity. (*b*) Two distinct conformations of the –PEt_3_ group present in this structure, viewed along (top) and perpendicular to (bottom) the Au—P bond.

In the originally published phase determined at 100 K, the triclinic crystal structure of pyrEt-α is based on a four-Au-atom-long rod [Fig. 1 ▸(*a*)]. The rods arrange along the [010], almost forming an infinite Au⋯Au chain in that direction, as described previously (Głodek *et al.*, 2018 ▸). Aurophilic interactions within the rod are quite short, whereas the distance between gold atoms from subsequent rods is over 4.4 Å, easily exceeding the sum of their van der Waals radii (3.4 Å). This general arrangement is retained at room temperature, with one important difference. Instead of two adjacent, distinct and perfectly ordered Au⋯Au chains, there is only one chain, with a disorder in the position of Au4 and the connected –PEt_3_ ligands. As a consequence, the room-temperature phase features a halved unit-cell parameter *a* compared with the 100 K conditions (Fig. S2 of the supporting information).

Note that the conformations of the triethylphosphine ligands at atoms Au2 and Au4 are distinct. They can be classified according to Orpen *et al.* (1998 ▸) and later Ellis *et al.* (2009 ▸) as F and G, accordingly [Fig. 1 ▸(*b*)]. Conformation F is the most commonly observed in the CCDC and features the lowest energy when considered individually (De Silva *et al.*, 2015 ▸). Conformation G is decidedly less energetically advantageous and rarer, though it has been observed in certain Au(I) compounds (Ellis *et al.*, 2009 ▸; De Silva *et al.*, 2015 ▸).

### 3.2. Evolution of unit-cell parameters and assigning critical pressure for phase transition

**Figure 2:**
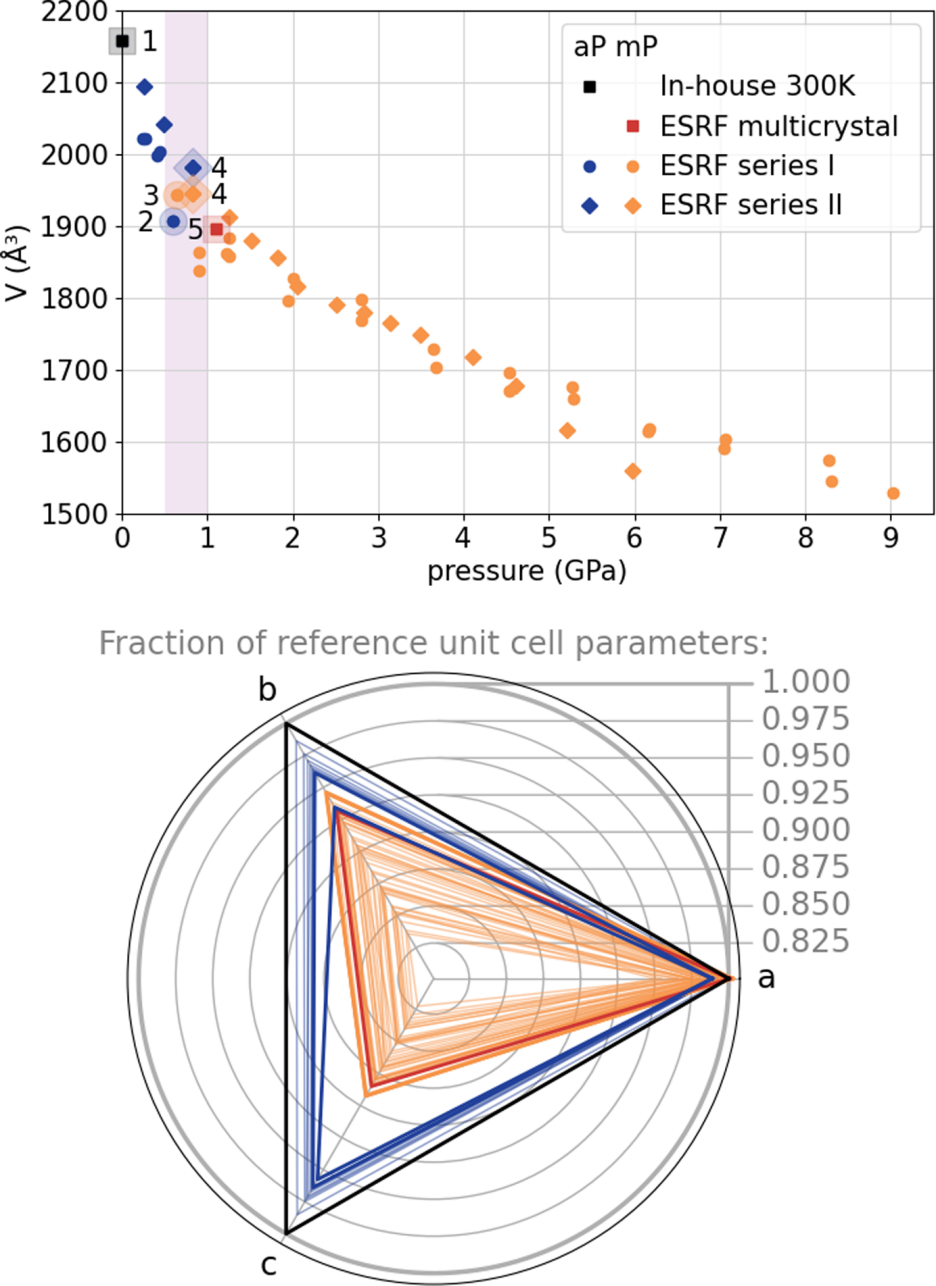
(Top) Evolution of the unit-cell volume of pyrEt-α with pressure. Data points referred to more specifically in this manuscript were highlighted and labeled with the crystallite number, according to Table 1 ▸. (Bottom) Graphical representation of the unit-cell-parameter contraction with pressure, represented as a normalized fraction of reference ambient-condition values on a ChARd plot. Each triangle represents one measured unit cell, complementing a single volume point shown on the left. The uncertainties on both graphs are negligible, not exceeding the point size on the left.

The phase transition from the triclinic space group *P*1 to monoclinic *P*2_1_/*c* is indicated at 0.65 (3) GPa in series I and 0.82 (3) GPa in series II (Fig. 2 ▸, top). All crystal specimens in the multi-crystal approach at 1.10 (3) GPa showed monoclinic unit-cell metrics and a systematic extinction pattern consistent with the presence of a *c*_[010]_ glide plane. The diffraction data from series I, collected with an amorphous Se detector at approximately 0.60 (3) GPa, were of noticeably lower resolution and generally too weak to permit an *ab initio* structure solution. This data inferiority, initially attributed to radiation damage, could stem from an ongoing structural reorganization of the crystal structure.

**Figure 3:**
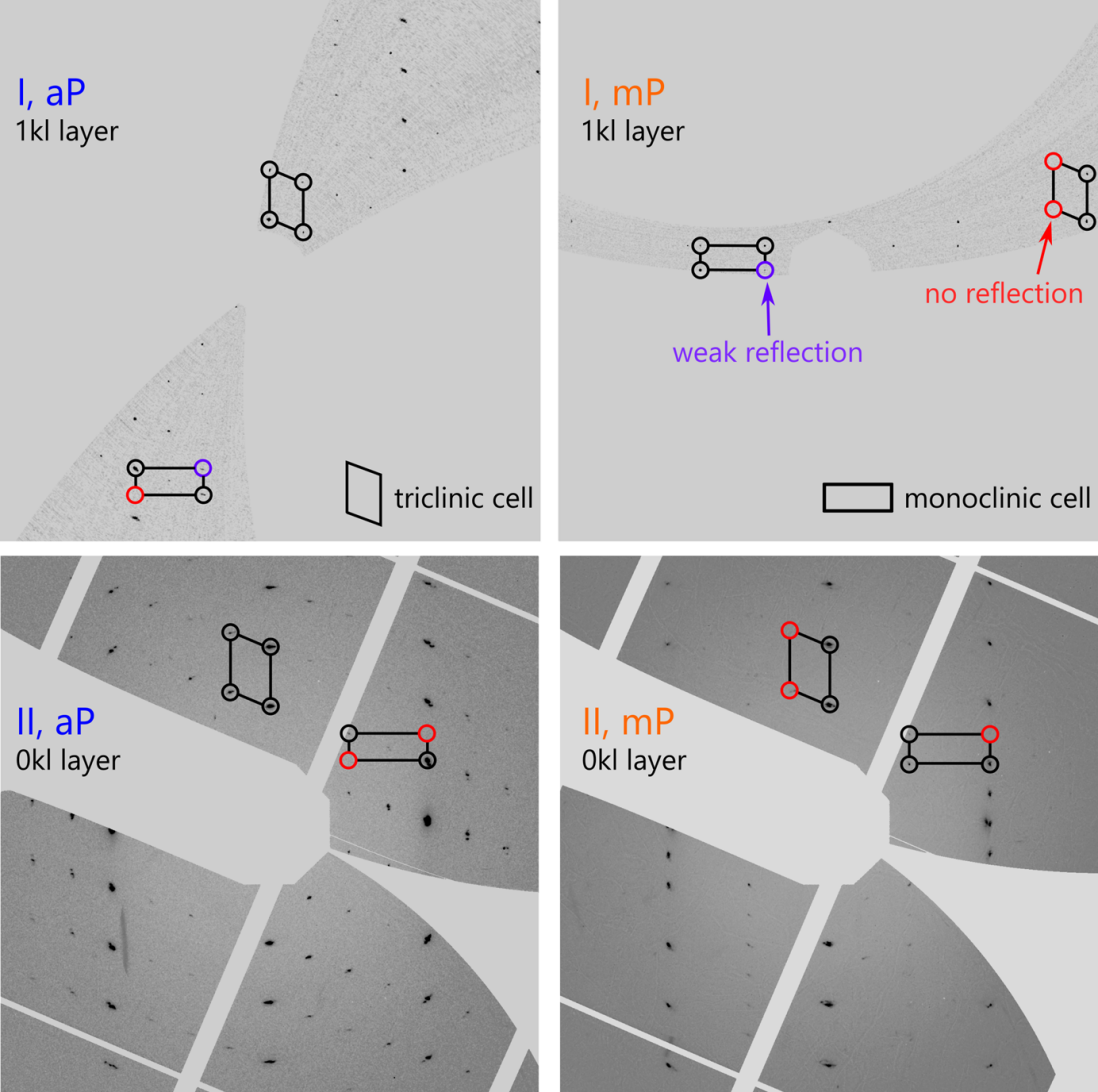
Reconstructions of selected reciprocal layers from datasets flanking the aP → mP phase transition from series I (*i.e.* crystals 2 and 3, upper row), and series II (*i.e.* crystal 4) before and after a 20 min interval (lower row). Red circles indicate where reflections expected for a given cell setting are missing. The patterns on the left index unambiguously in the triclinic unit cell, the patterns on the right in the monoclinic unit cell.

In order to illustrate the phase transition, unit-cell lengths *a*, *b* and *c* from individual experiments were normalized to the ambient-pressure reference and plotted in a radial coordinate system (Fig. 2 ▸, bottom). This approach was recently proposed by Kaźmierczak *et al.* (2021 ▸) and implemented in *matplotlib* for the purpose of this work (Tchoń, 2024 ▸). It allowed us to identify the 0.60 GPa series I data point collected on crystal 2 as an outlier on account of a skewed *a*:*b*:*c* ratio, deviating from both the triclinic and the monoclinic phases. Coupled with a streaky appearance of the diffraction spots (Fig. 3 ▸), it suggests that the sample was already undergoing the phase transition during the experiment. The dataset from crystal 2 in the same DAC collected 30 min later and at a slightly higher noted pressure of 0.65 (3) GPa indicated unambiguous monoclinic unit-cell metrics.

A pressurization sequence in the experimental series II yielded data points closest to the expected phase transition at 0.49 (3) and 0.82 (3) GPa. The dataset at the lower pressure indicated unambiguous triclinic symmetry. At higher pressure, the first dataset indicated also unequivocally a triclinic system, and the triclinic structure could be successfully refined based on this dataset starting from the room-temperature model (Table 1 ▸). A second dataset, collected within 20 min of the first from the same crystal specimen, yielded a pattern with monoclinic symmetry (Fig. 3 ▸) and allowed for *ab initio* structure solution in the monoclinic space group *P*2_1_/*c*. This result has been corroborated by the model obtained from unrestricted structure solution and refinement against high-coverage multi-crystal data collected at 1.10 (3) GPa.

Based on these observations, the actual onset of a pressure-induced phase transition can be pinpointed at ∼0.6 GPa. Pressurizing to about that point apparently pushed crystal 2 into a metastable state. Rapid data collection at the synchrotron allowed us to capture this state. In series II, rapid pressurization to ∼0.82 GPa appeared to ‘freeze’ crystal 4 in the triclinic phase with an un-contracted unit cell for the duration of data collection. Transformation to the monoclinic phase occurred in the next 20 min, leading to an apparently stable structure with a slightly contracted (*i.e.* more thermodynamically favorable) unit-cell volume.

### 3.3. Structural changes on phase transition

**Figure 4:**
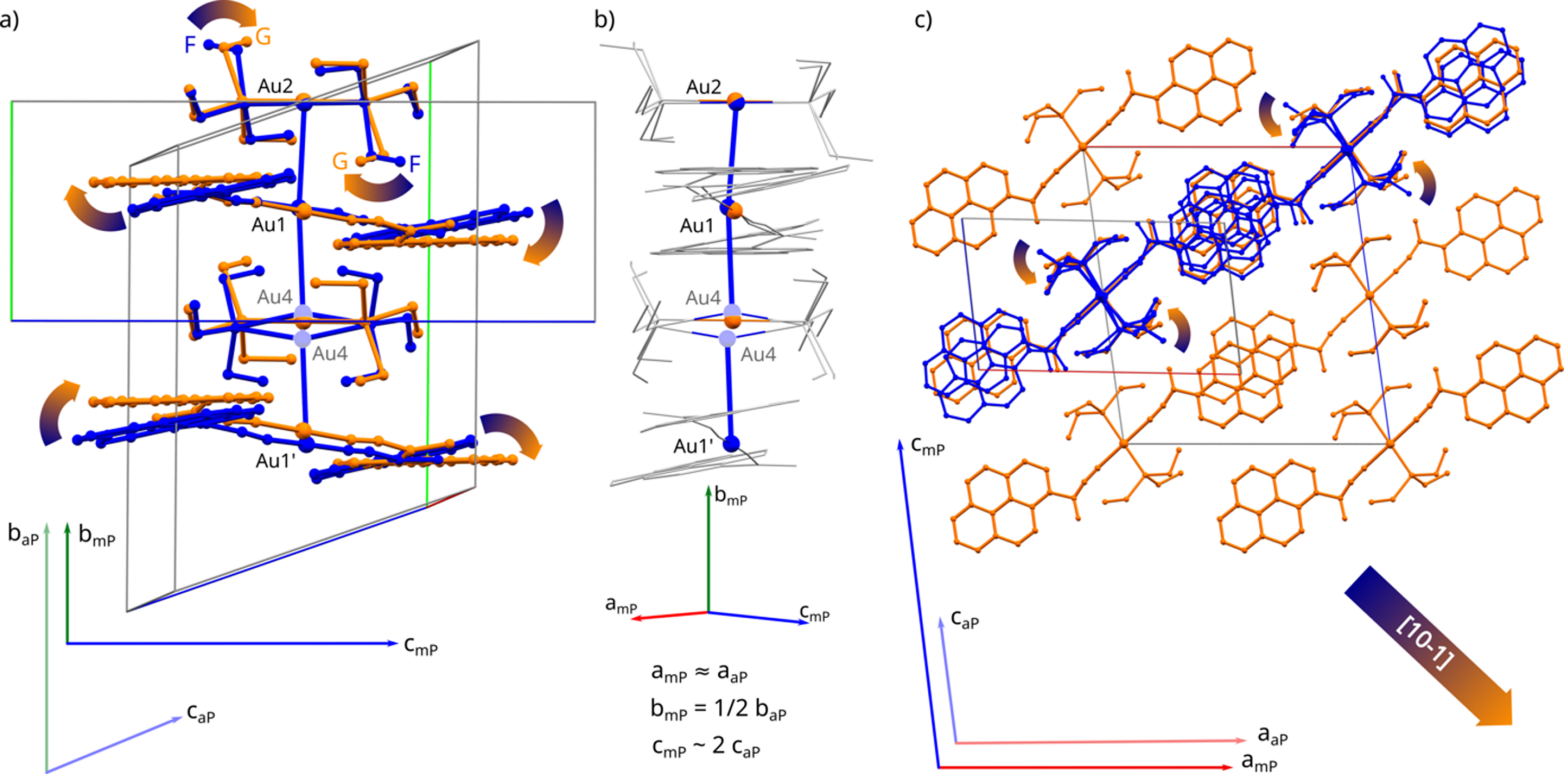
Overlay of crystal structures at 0.82 (3) GPa: triclinic (blue) and monoclinic (orange); conformational changes related to phase transitions are indicated with arrows. (*a*) Molecular motif of a 4Au rod. (*b*) Close-up of the Au⋯Au interactions; disorder of the Au4 atom from the triclinic system is being resolved. (*c*) Conformational change in the triethylphosphine leads to less steric hindrance in the [101] direction in the crystal lattice.

The phase transformation discovered can be categorized as displacive, yet it is accompanied by substantial structural changes. The appearance of the 2_1_ axis and *c* plane lowers the number of symmetry-independent pyrenyl and triethylphosphine moieties from 2 to 1 while preserving four pyrEt units per unit cell. Although the π-stacking arrangement of the pyrenyl moieties is retained, the inter-planar pyrene–pyrene distance becomes fixed at 1/2|**b**|. This new arrangement requires the pyrene moieties to recline away from [010] by 15°, making them almost perpendicular to the axis. The entire rotation can be viewed as lifting the adjacent columns along [010], bending the α angle to exactly 90° (Fig. 4 ▸).

In addition to the pyrene ring rotation, concerted rotations of a single ethyl group in adjacent molecular layers, analogous to formerly described motions for other metal–organic compounds, are observed (Makal, 2018 ▸). They reduce the inter-layer steric hindrance, leading to the relative shifts of layers in [101] direction. The conformation of all –PEt_3_ moieties switches from mixed F/G to uniformly G, rendering the new phase perfectly ordered.

Notably, though not unexpectedly (Wuttke *et al.*, 2018 ▸), the aurophilic interactions have the smallest impact in dictating the transformation. All gold atoms in the new *P*2_1_/*c* phase are located at inversion centers and evenly spaced along **b**, rendering the new phase fully polymeric. There is no tendency to preserve the shorter Au⋯Au contacts, as the average Au⋯Au distance within a ‘rod’ increases from 3.228 (6) to 3.365 (6) Å.

In the new crystal setting, the crystallographic direction [010] spanned by the Au⋯Au rods is preserved, but the length of the associated lattice vector **b** becomes halved. To accommodate the new symmetry, the [100] direction becomes perpendicular to [010], but otherwise undergoes little change. The new lattice vector **c** remains parallel to the pyrene plane but doubles in length and deviates from the original triclinic [001] by almost 30° (Figs. 4 ▸ and S2.1 of the supporting information).

The new higher-symmetry phase of pyrEt-α is a much more promising subject in the context of studying the structure–luminescence relationship. There remains just one variant of pyrene π-stacking and one variant of aurophilic interactions, both restricted by symmetry and bound with the **b** lattice constant. Nevertheless, our preliminary observations indicate that, while pyrEt-α displays significant pressure-induced red-shift in photoemission, the trend is not affected by the structural changes at ∼0.6 GPa. Detailed analysis of pyrEt-α luminescence as a function of temperature and pressure will be the subject of another publication.

### 3.4. Unrestrained crystal structure of pyrEt-α in the high-pressure phase

Crystal structure analysis under high pressure is particularly challenging for low-symmetry systems due to poor data coverage, which for a single crystal from a monoclinic system – most common in the Cambridge Structural Database (CSD) – may typically be well below 50%. This can hinder structure solution and bias the resulting crystal model, causing, for example, systematic shortening of certain bond lengths, necessitating the use of numerous constraints and strong restraints.

Among all the metal–organic structures determined at increased pressure and deposited in the CSD (Groom *et al.*, 2016 ▸) in the last four years, the typical data coverage for orthorhombic or lower symmetry systems is ∼40% up to the IUCr resolution limit of 0.82 Å (Spek, 2020 ▸) and only 12 structures among those contain more than 25 non-hydrogen atoms in the asymmetric unit.

In the context of our structure determination for pyrEt-α, the monoclinic phase is a positive outlier. The coverage obtained for a merged dataset was over 70% up to 0.65 Å, which allowed for unrestrained refinement of parameters pertaining to non-hyrogen atoms, yielding highly reliable molecular geometry. Using aspherical atomic scattering factors in the HAR approach proved to be fully justified, lowering the total discrepancy factor by over 2%. To the best of our knowledge, this is the largest low-symmetry system investigated structurally with quantum-crystallography tools.

## 4. Conclusions

We have successfully characterized a particular pressure-induced symmetry-increasing SCSC phase transition occurring in the crystal structure of a large metalorganic gold(I) compound (pyrEt) in its α crystal form by means of SCXRD. The modern instrumentation at the synchrotron facility have made it possible to collect (in series I) diffraction datasets confirming the coexistence of triclinic and monoclinic phases in two separate crystallites and with evident signs of increased strain, as a result, insufficient for structure determination. In another series (II), a similar experimental setup with a hybrid-pixel detector made it possible to collect two datasets from the same single-crystal specimen, with a 20 min delay, which allowed the structure determination of first pre-transition metastable triclinic and then post-transition stable monoclinic structures at the same pressure. As a result, details of pre- and post-transition structures and the steps necessary for structural reorganization could be captured for a large and labile compound at the moment of structural transformation.

Moreover, the structure of the new monoclinic phase was confirmed by data collected at 1.10 (3) GPa from multiple crystal specimens. A high-coverage merged dataset allowed unrestrained structure refinement with an aspherical atomic scattering factor formalism for this relatively large (>50 atoms per asymmetric unit) heavy-metal-containing organometallic compound.

The short span and quick succession of subsequent experiments that enabled us to observe the phase transition can yet be detrimental when it does not grant the crystal enough time to undergo a structural transformation. This puts in a different light the studies where certain phase transitions were theoretically predicted to occur at significantly lower pressures than reported based on XRD studies (Ukita *et al.*, 2016 ▸). Our example highlights how conceivable it is that in modern studies the experimentally determined phase-transition pressure can be sometimes overestimated, owing to the sparse sampling or rapid succession of subsequent diffraction experiments. A transition with particularly slow kinetics might even be totally overlooked. Ignoring the kinetics of a phase transformation would thus have serious consequences wherever the results of in-house diffraction experiments, typically spanning hours, were confronted with the outcomes of rapid synchrotron data collections, done in minutes. More importantly, it can have serious ramifications where parallel studies by X-ray and neutron diffraction have to be applied. With the recent advent in tools enabling high-pressure structural research with neutrons (Haberl *et al.*, 2023 ▸), the incompatibility of results may concern studies of ice, methane clathrates or metal superhydrides, as well as a number of magnetic materials consisting of large molecular building blocks. Our parting recommendation would be to perform repeated data collections at selected pressures, providing intervals of at least a few minutes, to detect the signs of potential transformations.

## Supplementary Material

Crystal structure: contains datablock(s) crystal_1, crystal_4a, crystal_4b, crystal_5. DOI: 10.1107/S2052252524007681/lt5070sup1.cif

Supporting figures and tables. DOI: 10.1107/S2052252524007681/lt5070sup2.pdf

CCDC references: 2360104, 2360105, 2360125, 2360126

## Figures and Tables

**Table 1:** Summary of the experiments performed for pyrEt-α (C_25_H_24_AuOP, 568.38 g mol^−1^) under atmospheric conditions and pressure points flanking the phase transition All experiments were performed at room temperature. The larger uncertainties and the missing structure-refinement statistics in series I result from the overall worse data quality; no structures could be refined for these series.

	In house 300 K	I		II		ESRF multicrystal
	Crystal 1	Crystal 2	Crystal 3	Crystal 4	Crystal 4	Crystal 5†
DAC	–	Membrane	Membrane	Membrane	Membrane	MB
PTM	–	He	He	He	He	Paratone
Pressure (GPa)	0.00001	0.60 (3)	0.65 (3)	0.82 (3)	0.82 (3)	1.10 (3)
*a* (Å)	16.0839 (5)	15.905 (8)	16.116 (15)	15.906 (7)	16.13 (4)	16.0966 (4)
*b* (Å)	14.2240 (7)	13.294 (18)	6.7243 (18)	13.677 (2)	6.7304 (9)	6.62840 (13)
*c* (Å)	10.1393 (3)	9.710 (6)	18.10 (7)	9.7733 (14)	18.096 (3)	17.9473 (14)
α (°)	109.816 (4)	110.32 (9)	90	109.591 (15)	90	90
β (°)	98.398 (2)	97.79 (5)	97.9 (2)	98.20 (2)	97.93 (8)	97.886 (6)
γ (°)	85.874 (3)	86.25 (7)	90	86.14 (3)	90	90
*V* (Å^3^)	2158.26 (15)	1907 (3)	1943 (8)	1982.2 (10)	1945 (5)	1896.77 (16)
*Z*, *Z*′	4, 2	4, 2	4, 1	4, 2	4, 1	4, 1
Space group	*P* 1	*P* 1	*P*2_1_/*c*	*P* 1	*P*2_1_/*c*	*P*2_1_/*c*
ρ_calc_ (g cm^−3^)	1.749	1.980	1.943	1.905	1.941	1.990
Wavelength (Å)	0.71073	0.41097	0.41097	0.41004	0.41004	0.41097
Unique reflections	13173	3412	1310	3120	2004	3932
*R*_int_	0.031	0.239	0.198	0.046	0.163	0.059
Resolution (Å)	0.70	0.51	0.79	0.56	0.56	0.59
Completeness	0.998	0.239	0.292	0.254	0.306	0.714
Reflections, restraints, parameters	42542, 32, 603	NA	NA	4078, 279, 263	3884, 632, 235	3932, 0, 256
*R*_1_ [*I*> 2σ(*I*)]	0.039	NA	NA	0.122	0.172	0.055
*wR*_2_ [*I*> 2σ(*I*)]	0.071	NA	NA	0.271	0.374	0.174
*R*_1_ (all data)	0.070	NA	NA	0.324	0.411	0.079
*wR*_2_ (all data)	0.081	NA	NA	0.367	0.483	0.229
Δρ_min/max_ (eÅ^−3^)	−1.472/1.011	NA	NA	−0.772/1.088	−0.910/1.145	−2.104/2.432

† Three distinctly oriented pieces of the same single crystal.
