# Supplementary material for: Structure–property relationship of a complex photoluminescent arylacetylide-gold(I) compound. I: a pressure-induced phase transformation caught in the act
Source: IUCrJ. 2024 Aug 23;11(Pt 5):737–43. doi: 10.1107/S2052252524007681 (PMC11364033; doi:10.1107/S2052252524007681)
Supplement: Supplementary file 2 [file m-11-00737-sup2.pdf]

IUCrJ

Volume 11 (2024)

Supporting information for article:

Structure-properties of a complex photoluminescent arylacetylide-gold(I) compound I: pressure-induced phase transformation caught in the act

Róża Jastrzębska, Tomasz Poręba, Federico Cova, Daniel M. Tchoń and Anna Makal

Structure-properties of a complex photoluminescent arylacetylide-gold(I) compound I: Pressure-induced phase transformation caught in-the-act Supporting Information

Róża Jastrzębska, Tomasz Poręba, Federico Cova,
Daniel Tchoń and Anna Makal
contact: *DTchon@lbl.gov,am.makal@uw.edu.pl*

July 30, 2024

Contents

S1 X-ray Diffraction **S2**
S1.1 Crystals placement in the DAC-s S2
S1.2 Unit Cell constants determined in membrane-DAC experiments . . . S3

S2 Comparisons of Crystal Structures **S4**
S2.1 300K vs the originally deposited structure S4
S2.2 Structure of pyrEt mP determined at 1.10 GPa S6
S2.3 Structures determined at the point of phase transition S6
S2.4 CCDC deposition numbers S9

S3 References **S9**

S1 X-ray Diffraction

S1.1 Crystals placement in the DAC-s

In the first high-pressure data collection series (I), four crystal specimens of pyrEt were placed in a membrane DAC of BX90-type (cullet diameter 600μm, gasket hole 300μm, gasket thickness 100μm, 2θ OA 32°) with gaseous He as a PTM. The objectives were: to gain more data coverage by collecting diffraction data from two specimens at each pressure point, and to compensate for radiation damage (Figure S1.1 left). The first objective was not achieved, as specimens turned out to be all in similar orientations, with the same main face on a cullet. These specimens yielded a total of 29 useful datasets pressure range from 0.25 GPa to 2.0 GPa (crystals 2 and 3) and then up to 9.03 GP (the remaining two specimens) Two datasets, at pressure of 0.61 (crystal 2) and 0.65 GPa (crystal 3) accordingly, are commented in more detail. In the second series, a single crystal specimen (crystal 4) was placed in a membrane DAC of the same design alongside single crystals of three other substances (Figure S1.1 middle). It yielded a total of 16 useful datasets in pressure range from 0.25 GPa to 5.98 GPa, two of which were collected 20 minutes apart at the same pressure of 0.82 GPa.

In order to increase the reciprocal space coverage for the new crystal phase and facilitate unrestrained structure refinement, a total of 5 larger pieces cut out of a single crystal 5 of pyrEt were placed in a single DAC of a Merill-Basset design in various orientations (cullet diameter 750μm, gasket hole 400μm, gasket thickness 200μm, 2θ OA 40°) with ParatoneN oil as PTM - the only liquid PTM available on site just then which would not dissolve our sample. As its composition is the closest to the recognized Fluorinert or Vaseline oils, it is safe to assume that ParatoneN can still provide quasi-hydrostatic conditions at 1.1 GPa, although it would be highly unsuitable for structural studies in a wider pressure range.(Klotz, Chervin, Munsch & Le Marchand 2009, Tateiwa & Haga 2009)

Useful diffraction datasets were collected from the three largest pieces (Figure S1.1 right).

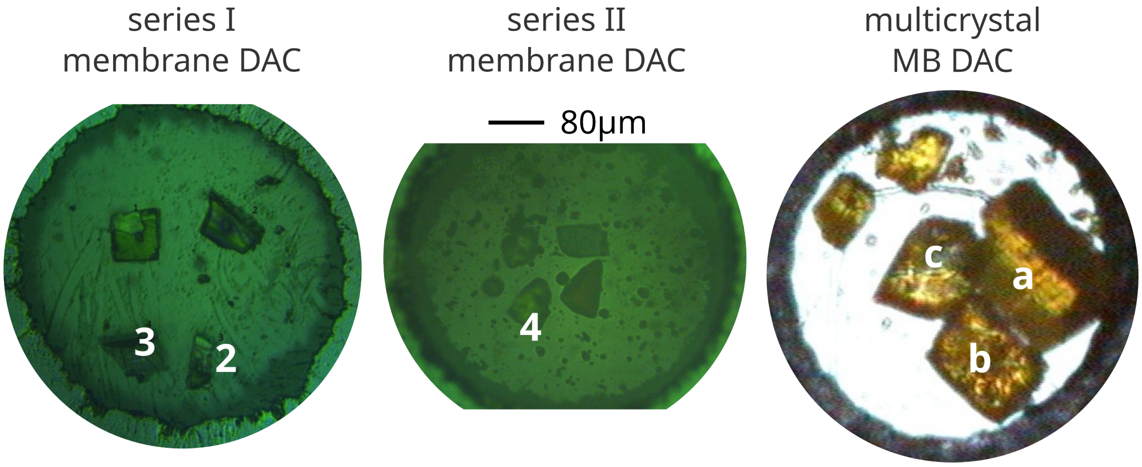

Figure S1.1: *Crystal specimens placed in a DAC in high-pressure experimental series. Specimens discussed in details in this work are numbered in white. Three pieces of Crystal 5 in the multi-crystal experiment marked tentatively as a, b and c. Radiation damage marks visible as darker spots on the surfaces of crystals in series I.*

S1.2 Unit Cell constants determined in membrane-DAC experiments

Table S1.1: *Unit cell constants obtained in variable-pressure experiments series I and II. Data suitable for structure refinements.*

Pressure [GPa]	1.10(5)	1.94(5)	3.64(5)	5.27(5)	9.03(5)
Phase	mP	mP	mP	mP	mP
Space Group	P 1 21/c 1	P 1 21/c 1	P 1 21/c 1	P 1 21/c 1	P 1 21/c 1
a [Å]	16.0966(4)	15.9590(7)	15.8982(16)	15.843(2)	15.5233(9)
b [Å]	6.62840(13)	6.4376(13)	6.29510(13)	6.1903(4)	5.94620(15)
c [Å]	17.9473(14)	17.652(2)	17.438(5)	17.252(15)	16.681(5)
α [°]	90	90	90	90	90
β [°]	97.886(6)	98.013(7)	97.968(15)	97.59(4)	96.772(16)
γ [°]	90	90	90	90	90
Volume [Å^3]	1896.77(16)	1795.8(5)	1728.4(5)	1677.1(15)	1529.0(5)
Reflections #	3740	2061	2795	2685	2239
R_{int}	0.0590	0.0421	0.0433	0.0682	0.0266
Resolution [Å]	0.5986	0.7802	0.7801	0.7809	0.7705
Completeness	0.714	0.364	0.313	0.312	0.284

Pressure [GPa]	0.26(5)	0.49(5)	0.82(5)	0.82(5)	1.83(5)
Phase	aP	aP	aP	mP	mP
Space Group	P -1	P -1	P -1	P 1 21/c 1	P 1 21/c 1
a [Å]	16.044(4)	15.955(11)	15.906(7)	16.13(4)	16.04(3)
b [Å]	14.0312(8)	13.887(4)	13.677(2)	6.7304(9)	6.5563(8)
c [Å]	9.9880(5)	9.885(2)	9.7733(14)	18.096(3)	17.828(2)
α [°]	109.677(5)	109.58(3)	109.591(15)	90	90
β [°]	98.252(10)	98.41(4)	98.20(2)	97.93(8)	98.01(6)
γ [°]	86.203(11)	86.78(5)	86.14(3)	90	90
Volume [Å^3]	2094.9(6)	2041.3(16)	1982.2(10)	1945(5)	1857(4)
Reflections #	3927	3739	4078	3884	3495
R_{int}	0.0208	0.0335	0.0464	0.1625	0.1239
Resolution [Å]	0.5723	0.5696	0.5623	0.5600	0.5735
Completeness	0.231	0.233	0.254	0.306	0.295

Pressure [GPa]	2.84(5)	3.49(5)	4.11(5)
Phase	mP	mP	mP
Space Group	P 1 21/c 1	P 1 21/c 1	P 1 21/c 1
a [Å]	16.00(2)	15.918(17)	15.870(12)
b [Å]	6.3929(5)	6.3402(5)	6.2828(2)
c [Å]	17.5682(13)	17.4962(12)	17.3915(8)
α [°]	90	90	90
β [°]	98.00(3)	98.01(3)	97.93(2)
γ [°]	90	90	90
Volume [Å^3]	1779(2)	1748.6(18)	1717.5(13)
Reflections #	3552	3557	2525
R_{int}	0.1011	0.0563	0.1225
Resolution [Å]	0.5752	0.5709	0.5677
Completeness	0.301	0.307	0.215

S2 Comparisons of Crystal Structures

S2.1 300K vs the originally deposited structure

In the originally published phase (Głodek, Makal, Paluch, Kądziołka-Gaweł, Kobayashi, Zakrzewski & Plażuk 2018) determined at 100 K, the triclinic crystal structure of pyrEt is based on a 4-Au-atom-long rod. The rods arrange along the [010] direction, almost, but not fully, forming and infinite Au$\cdots$Au chain in that direction. Aurophilic interactions Au$\cdots$Au within the rod are quite short, while the 'external' Au$\cdots$Au distance is significantly longer, over 4.4 Å. This general arrangement is retained at room temperature, with one important difference. Instead of two adjacent, distinct and perfectly ordered Au$\cdots$Au chains there is only one chain, with a disorder in the position of Au4 and the connected –PEt_3 ligands. As a consequence,

Table S1.2: *Unit cell constants obtained in variable-pressure experiments series I.*

Pressure [GPa]	0.24(5)	0.27(5)	0.42(5)	0.45(5)	0.60(5)	0.65(5)
Phase	aP	aP	aP	aP	aP	mP
Space Group	P -1	P -1	P -1	P -1	P -1	P 1 21/c 1
a [Å]	15.957(3)	15.948(5)	15.926(2)	15.955(4)	15.905(8)	16.116(15)
b [Å]	13.763(5)	13.8134(12)	13.708(3)	13.7282(13)	13.294(18)	6.7243(18)
c [Å]	9.862(2)	9.842(4)	9.8078(13)	9.790(4)	9.710(6)	18.10(7)
α [°]	109.33(3)	109.55(2)	109.406(16)	109.29(2)	110.32(9)	90.0
β [°]	98.312(17)	98.15(3)	98.142(11)	98.13(3)	97.79(5)	97.9(2)
γ [°]	86.22(2)	86.221(19)	86.276(14)	86.327(15)	86.25(7)	90.0
Volume [Å^3]	2022.1(9)	2022(1)	1998.8(6)	2003.2(10)	1907(3)	1943(8)
Reflections #	5587	5560	5946	5547	5008	3011
R_{int}	0.0756	0.1070	0.0781	0.1107	0.2391	0.1975
Resolution [Å]	0.5204	0.5307	0.5168	0.5289	0.5149	0.7917
Completeness	0.1230	0.1300	0.1216	0.1292	0.1170	0.2925

Pressure [GPa]	0.90(5)	0.91(5)	1.22(5)	1.25(5)	1.26(5)	2.00(5)
Phase	mP	mP	mP	mP	mP	mP
Space Group	P 1 21/c 1	P -1	P 1 21/c 1	P 1 21/c 1	P 1 21/c 1	P 1 21/c 1
a [Å]	15.9942(11)	15.887(14)	16.071(5)	16.117(7)	16.0528(11)	16.075(2)
b [Å]	6.5974(12)	6.582(3)	6.577(3)	6.5930(9)	6.5674(2)	6.4869(5)
c [Å]	17.826(2)	17.692(17)	17.885(8)	17.90(2)	17.796(6)	17.689(13)
α [°]	90.0	90.46(10)	89.66(4)	90.0	90.0	90.0
β [°]	97.878(8)	97.72(8)	98.08(3)	98.02(9)	97.879(19)	97.92(4)
γ [°]	90.0	89.08(8)	90.20(3)	90.0	90.0	90.0
Volume [Å^3]	1863.3(4)	1833(3)	1872(1)	1884(3)	1858.5(7)	1827(1)
Z	4	4	4	4	4	4
Reflections #	2931	3040	2922	3014	3053	2225
R_{int}	0.0641	0.3950	0.0750	0.0886	0.0403	0.0455
Resolution [Å]	0.7801	0.7800	0.7803	0.7800	0.7805	0.7812
Completeness	0.2229	0.1466	0.2212	0.1536	0.1911	0.1946

Pressure [GPa]	2.81(5)	2.81(5)	3.68(5)	4.53(5)	4.53(5)	5.29(5)
Phase	mP	mP	mP	mP	mP	mP
Space Group	P 1 21/c 1	P 1 21/n 1	P 1 21/c 1	P 1 21/c 1	P 1 21/c 1	P 1 21/c 1
a [Å]	16.15(3)	16.066(17)	15.81(2)	15.777(4)	15.869(2)	15.785(10)
b [Å]	6.518(8)	6.381(3)	6.241(3)	6.2018(8)	6.2416(4)	6.1728(14)
c [Å]	17.3(2)	17.28(11)	17.41(12)	17.23(3)	17.287(11)	17.20(2)
α [°]	90.0	90.0	90.0	90.0	90.0	90.0
β [°]	96.5(6)	98.0(3)	98.4(3)	97.77(7)	97.66(3)	97.57(8)
γ [°]	90.0	90.0	90.0	90.0	90.0	90.0
Volume [Å^3]	1804(20)	1754(11)	1699(12)	1671(3)	1697(1)	1661(3)
Reflections #	2818	2828	2663	2638	2717	2693
R_{int}	1.0270	0.9589	0.1742	0.1370	0.1341	0.5104
Resolution [Å]	0.7801	0.7824	0.7801	0.7801	0.7803	0.7801
Completeness	0.1565	0.1845	0.1905	0.1489	0.2084	0.1987

Pressure [GPa]	6.16(5)	6.17(5)	7.05(5)	7.07(5)	8.27(5)	8.31(5)
Phase	mP	mP	mP	mP	mP	mP
Space Group	P 1 21/c 1	P 1 21/c 1	P 1 21/c 1	P 1 21/c 1	P 1 21/c 1	P 1 21/c 1
a [Å]	15.6783(14)	15.6976(9)	15.6463(13)	15.6862(15)	15.6327(13)	15.532(7)
b [Å]	6.0957(3)	6.10023(13)	6.0529(3)	6.0683(2)	6.0141(2)	5.9644(6)
c [Å]	17.025(10)	17.031(5)	16.929(11)	16.970(9)	16.873(8)	16.81(2)
α [°]	90.0	90.0	90.0	90.0	90.0	90.0
β [°]	97.28(3)	97.343(16)	97.07(3)	97.07(3)	96.92(2)	97.24(9)
γ [°]	90.0	90.0	90.0	90.0	90.0	90.0
Volume [Å^3]	1614(1)	1617.5(5)	1591(1)	1603.0(9)	1574.8(8)	1545(2)
Z	4	4	4	4	4	4
Reflections #	2562	2607	2550	2600	2507	2533
R_{int}	0.0901	0.0624	0.0830	0.0532	0.1036	0.1148
Resolution [Å]	0.7808	0.7813	0.7814	0.7809	0.7802	0.7800
Completeness	0.1523	0.3068	0.2874	0.3043	0.2835	0.3111

the RT phase displays twice shorter **a** unit cell constant than at 100K (Figure S2.1).

Table S1.3: *Unit cell constants obtained in variable-pressure experiments series II.*

Pressure [GPa]	1.25(5)	1.52(5)	2.05(5)	2.51(5)	4.61(5)	5.21(5)	5.98(5)
Opening angle [°]							
Phase	mP	mP	mP	mP	mP	mP	mP
Space Group	P 1 21/c 1	P 1 21/c 1	P 1 21/c 1	P 1 21/c 1	P 1 21/c 1	P 1 21/c 1	P 1 21/c 1
a [Å]	16.01(4)	16.041(18)	16.02(8)	15.92(6)	15.87(4)	15.35(6)	15.28(8)
b [Å]	6.6757(15)	6.6063(5)	6.468(2)	6.4344(15)	6.2028(9)	6.1711(15)	6.0661(16)
c [Å]	18.063(4)	17.9103(13)	17.720(8)	17.655(5)	17.214(3)	17.227(4)	16.995(6)
α [°]	90.0	90.0	90.0	90.0	90.0	90.0	90.0
β [°]	97.95(7)	97.91(3)	98.29(15)	98.07(11)	97.95(7)	97.88(11)	97.90(16)
γ [°]	90.0	90.0	90.0	90.0	90.0	90.0	90.0
Volume [Å^3]	1913(5)	1880(2)	1817(10)	1790(7)	1678(4)	1616(6)	1560(8)
Reflections collected	1098	4137	3905	3919	3306	3184	2978
R_{int}	0.1073	0.1475	0.4168	0.3076	0.2720	0.3928	0.5754
Resolution [Å]	1.1445	0.5760	0.5698	0.5687	0.5607	0.5669	0.5575
Completeness	0.1167	0.1895	0.1823	0.1825	0.1633	0.1688	0.1569

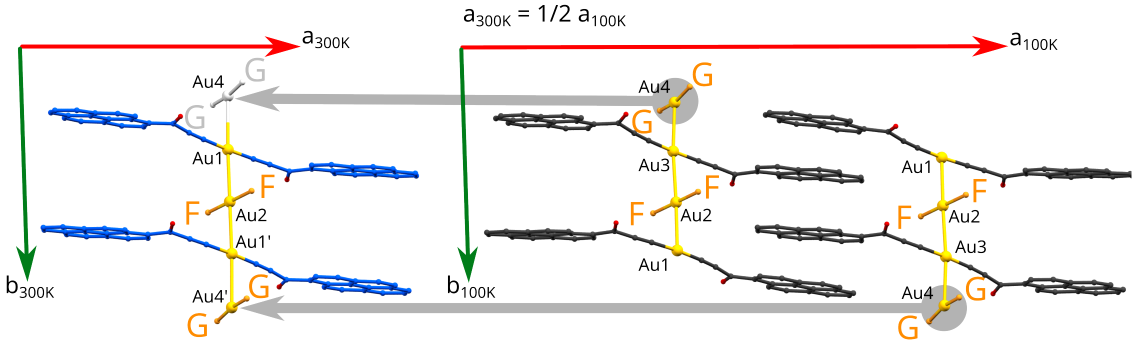

Figure S2.1: *Crystal structure of pyrEt determined at 1 atm and 300K (C atoms blue) in relation to the formerly described structure at 100K (C atoms dark-grey). View along [001] in triclinic cell setting. H atom positions omitted for clarity, as well as –Et$_3$ groups, here schematically represented as F or G, depending on the conformation. The alternative position of atom Au4 with associated phosphines represented in light-grey.*

S2.2 Structure of pyrEt mP determined at 1.10 GPa

S2.3 Structures determined at the point of phase transition

The crystal structures of pyrEt aP and pyrEt mP determined in experimental series II from crystal 4 were necessarily refined with substantial number of restraints and constraints. This was due to: a) low completeness of diffraction data, resulting directly from placing plate-like crystals in a bulky DAC on one of their own faces and b) the lowered data quality due to experiment being conducted at the point of phase transformation. The general reasonableness of the obtained models was confirmed by comparing the structures of II-aP and II-mP with the structures determined at 300K at 1 atm or at 1.10 GPa in the course of unrestrained refinements. Figure S2.3 illustrates the structural overlays of the main structural motifs of the II-aP against the 300K and accordingly II-mP at 1.10 GPa. In both instances the RMS deviation as calculated by Mercury (Macrae, Bruno, Chisholm, Edgington, McCabe, Pidcock, Rodriguez-Monge, Taylor, van de Streek & Wood 2008) based on non-H atoms was $\approx$ 0.17 Å, indicating substantial structural agreement. It is worth noting, that in

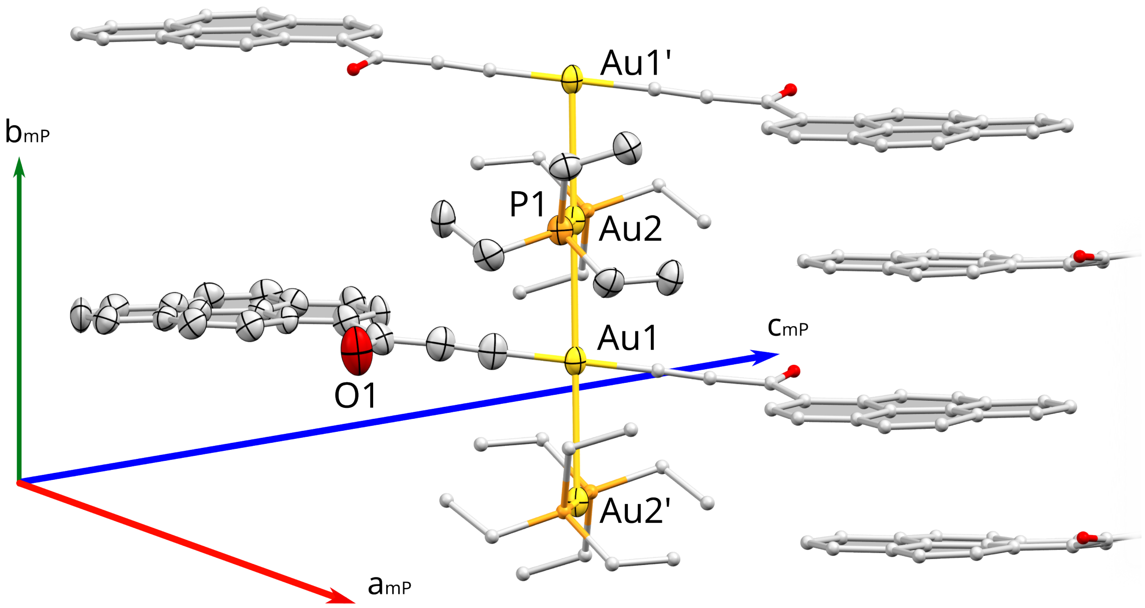

Figure S2.2: *(a) The main building block of the crystal structure of pyrEt mP at 1.10 GPa: an infinite chain of Au···Au atoms along [010] direction. Atomic displacement parameters presented for atoms from asymmetric unit only at 50 % probability. H atom positions omitted for clarity.*

case of II-aP against the 300K comparison, the conformations of triethylphosphines at Au4 site differ in the direction of the bend in one of the –Et groups, although they still qualify as G.

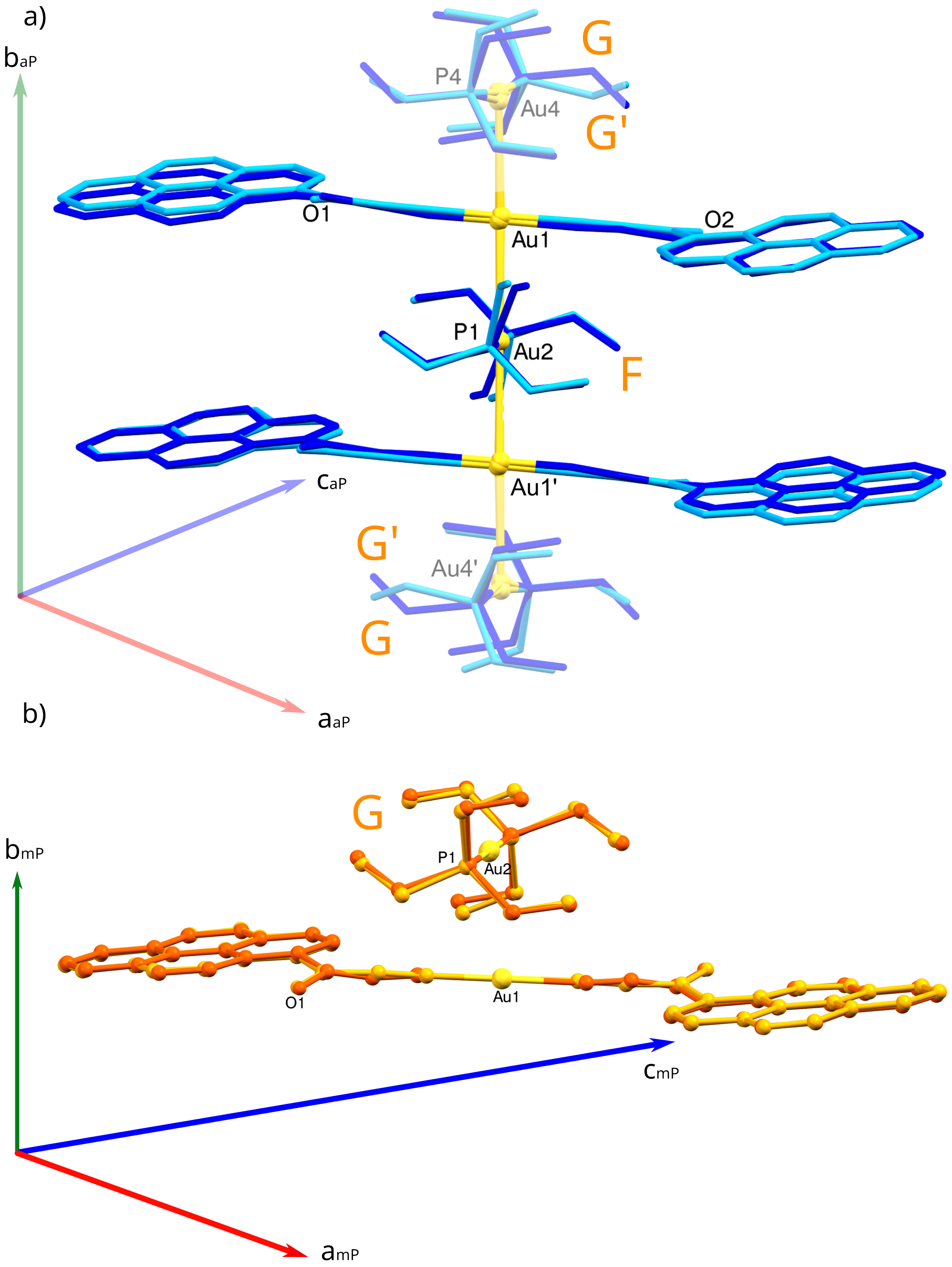

Figure S2.3: *Comparison of the main structural motifs in (a) the pyrEt aP phase, comparing the II-aP structure (dark blue) against structure determined at 300K at 1 atm (cyan) and (b) the pyrEt mP phase comparing the II-aP structure (orange) against structure determined at 293K at 1.10 GPa (light orange).*

S2.4 CCDC deposition numbers

Structures determined for the purpose of this paper were deposited as individual entries within Cambridge Structural Database(Groom, Bruno, Lightfoot & Ward 2016). Exact deposition number for each structure have been presented in Table S2.1.

Table S2.1: *CCDC deposition numbers of crystal structures determined for the purpose of this work, sorted by crystal phase and exerted pressure.*

Data	phase	T	Pressure	CCDC deposition number
crystal 1	aP	300K	atmospheric	2360105
II crystal 4	aP	293K	0.82 GPa	2360125
II crystal 4	mP	293K	0.82 GPa	2360126
crystal 5	mP	293K	1.10 GPa	2360104

S3 References

Głodek, M., Makal, A., Paluch, P., Kądziołka-Gaweł, M., Kobayashi, Y., Zakrzewski, J. & Plażuk, D. (2018). *Dalton Transactions*, 47(19), 6702–6712.

Groom, C. R., Bruno, I. J., Lightfoot, M. P. & Ward, S. C. (2016). *Acta Crystallographica Section B*, 72(2), 171–179.

Klotz, S., Chervin, J.-C., Munsch, P. & Le Marchand, G. (2009). *Journal of Physics D: Applied Physics*, 42(7), 075413.

Macrae, C. F., Bruno, I. J., Chisholm, J. A., Edgington, P. R., McCabe, P., Pidcock, E., Rodriguez-Monge, L., Taylor, R., van de Streek, J. & Wood, P. A. (2008). 41(2), 466–470.

Tateiwa, N. & Haga, Y. (2009). *Review of Scientific Instruments*, 80(12), 123901.
